# Exploring Stroke Patients’ Needs: A Cultural Adaptation and Validation of the Modified Needs Assessment Questionnaire in a Greek Context

**DOI:** 10.3390/healthcare12131274

**Published:** 2024-06-26

**Authors:** Katerina Paschalidou, Efi Tsitskari, Anna Tsiakiri, Evangelia Makri, Pinelopi Vlotinou, Konstantinos Vadikolias, Nikolaos Aggelousis

**Affiliations:** 1 Department of Physical Education and Sport Science, Democritus University of Thrace, 69100 Komotini, Greece; kapascha@phyed.duth.gr (K.P.); etsitska@phyed.duth.gr (E.T.); emakri@phyed.duth.gr (E.M.); nagelous@phyed.duth.gr (N.A.); 2 Department of Neurology, School of Medicine, Democritus University of Thrace, 68100 Alexandroupolis, Greece; kvadikol@med.duth.gr; 3 Department of Occupational Therapy, University of West Attica, 12243 Athens, Greece; pvlotinou@uniwa.gr

**Keywords:** post-stroke care, needs assessment, validity, rehabilitation, quality of life

## Abstract

Stroke survivors often face diverse unmet needs highlighting the significance of identifying and addressing these needs to enhance rehabilitation outcomes and overall quality of life. This study aimed to validate the modified Needs Assessment Questionnaire (mNAQ) as a reliable and valid tool for assessing the needs of stroke patients in the Greek context. Additionally the research sought to identify potential differences in the assessment of stroke patients’ needs based on their stroke phase and National Institutes of Health Stroke Scale (NIHSS) scores. A sample of 71 adult stroke survivors adhering to World Health Organization guidelines and providing autonomous consent participated in the study. The mNAQ comprising 141 items across 12 domains was utilized to evaluate stroke patients’ needs. The NIHSS and Barthel Index (BI) were employed for functional independence and mobility assessment. Data analysis incorporated confirmatory factor analysis, exploratory factor analysis and Cronbach’s reliability analysis to establish construct validity and internal consistency. Concurrent and known-groups validity analyses were conducted; and Spearman’s rho correlation explored the relationship between mNAQ and BI scores. Non-parametric analyses were applied to identify differences based on stroke phase and NIHSS scores. The study revealed that the mNAQ initially lacked satisfactory psychometric properties in the Greek context. Subsequent modifications guided by confirmatory and exploratory factor analyses resulted in a refined three-factor scale encompassing 31 items in the domains of communication, mobility, and social functioning needs. This adapted measure effectively differentiated between acute and chronic stroke patients and those with minor and moderate strokes. In conclusion, the validated 31-item Greek mNAQ emerges as a crucial tool for comprehensively assessing the needs of stroke patients. Its application holds promise for optimizing post-stroke care improving functional outcomes and ultimately enhancing the overall well-being and quality of life for stroke survivors.

## 1. Introduction

The exploration of the link between the users’ perceptions, preferences, expectations, and satisfaction with care provides valuable information regarding deficiencies from the ideal care close to a patient’s needs [1]. Previous research demonstrates that patients’ needs & expectations are not receiving much attention, specifically the needs and expectations of stroke patients, in addition to the scarce investigation of factors influencing their unmet needs [2].

Zawawi, Aziz, Fisher, Ahmad & Walker [3] through a systematic review, concluded to a wide range of unmet needs in stroke survivors living their life after stroke, including information provision, financial support for caregivers, community support, physical symptoms, rehabilitation services, psychological, transfer & mobility, social/recreational, employment, self-care, home management, and social interaction. Although the abovementioned factors’ associations are found to be inconsistent and the differences between them are usually due to the research design (such as the timing that the needs are captured, the instruments that are used, or the population that is studied), sharing knowledge about unmet needs may reduce fragmentation in stroke care, contribute to a sustainable and dynamic stroke care delivery and encourage optimal use of resources available [3]. 

Previous studies suggest that self-perceived needs assessment can help the rehabilitation process by supporting autonomy, social connection, and adaptation [4], focusing on functional performance and self-management [5], and addressing concerns related to self-perceived health and daily activities [6]. The long-term problems experienced by stroke survivors can significantly decrease their quality of life. Additionally, the different phases of stroke, including the acute, subacute, and long-term or chronic phases, play a crucial role in determining the evolving rehabilitation needs of stroke patients, with each phase requiring tailored interventions to maximize functional recovery, address specific impairments, and support social reintegration and participation in daily activities [7]. By evaluating and reviewing the needs of stroke patients, healthcare providers and policymakers can identify gaps in care and develop targeted interventions to improve the overall quality of life for stroke patients.

Several studies have examined the interplay between stroke severity, the phases of stroke, and rehabilitation outcomes, shedding light on the varying needs of stroke patients at different stages of their recovery journey. For instance, Kwakkel et al. [8] investigated the relationship between stroke severity and rehabilitation outcomes, revealing that patients with more severe strokes, as indicated by higher NIHSS scores, experienced lower functional outcomes and longer rehabilitation stays. These findings underscore the necessity for more intensive and specialized rehabilitation interventions to address the complex motor and cognitive impairments of these patients. 

Kristensen et al. [9] found that people with severe disability after a stroke tend to report more unmet needs for rehabilitation and are less satisfied than people with mild to moderate disability [10]. Additionally, Hatem et al. [11] explored the combined impact of stroke severity and phases on rehabilitation needs, revealing that patients with severe strokes faced more substantial challenges across all phases of recovery, necessitating more intensive therapy and longer rehabilitation stays. Notably, specific needs, such as communication and emotional support, were accentuated during the subacute phase, emphasizing the importance of tailored interventions to meet evolving requirements throughout the recovery journey [12]. Phases refer to Acute stroke that is characterized by a sudden onset of symptoms that persist for more than 24 h, with the critical treatment window being within the first 6 h. Chronic stroke refers to the period following the acute phase, focusing on long-term recovery [8]. Understanding these time definitions is essential for timely and effective stroke management.

All the above, illustrate how stroke severity and the phases of stroke significantly influence rehabilitation needs and outcomes. Therefore, to assess the needs of people with stroke, there should be a valid tool for recording them, which may be used by health professionals (doctors, nurses, physiotherapists, exercise trainers) in order to contribute to the better management and rehabilitation of the patient. The modified Needs Assessment Questionnaire (mNAQ) comprised 141 items in 12 domains and was developed by Olaleye et al. [13] to assess expectations of patients with neurological conditions (NCs) from rehabilitation. 

The aim of this research was to validate the mNAQ as a dependable and accurate tool to assess the needs of stroke patients in a Greek setting and further investigate whether they differentiate in terms of the stroke severity and the phase of the stroke. 

This research is justified by the need to address the gap in assessing and addressing the unmet needs of stroke patients, which can lead to more effective rehabilitation strategies and improved patient outcomes.

The organization of this research article is as follows: Section 2 provides a detailed description of the methodology used in this study. Section 3 presents the results of the validation of the mNAQ in the Greek setting. Section 4 discusses the findings in the context of existing literature and their implications for stroke rehabilitation. Finally, Section 5 concludes the paper by summarizing the key findings and Section 6 is suggesting directions for future research.

## 2. Materials and Methods

### 2.1. Sample & Data Collection

Individuals who met the following criteria were eligible to participate in the study: (1) being an adult, (2) having received a diagnosis of stroke according to the World Health Organization guidelines, and (3) having the capability to give consent autonomously. The sample consisted of 71 stroke patients in acute/subacute phase (79%) and in chronic phase (21%), mostly men (63.4%), aged 60–69 (42.3%). Seventy-eight percent (78%) of them had a minor stroke and 22% moderate stroke. All participants were diagnosed with ischemic stroke and were adults (18 years old and older). Sample demographics appear in the Results section.

### 2.2. Measures

Olaleye, et al. [13] produced a survey consisting of 141 items to evaluate the requirements of stroke patients. The survey included 12 themes. The questionnaire [13] was adjusted and tailored for a Greek patient group after we received permission from the researchers to utilize it. Back-to-back translation was performed [14]. First, two of the authors translated the original scale into Greek and compared the two versions. One hundred and two (102) of the one hundred and forty-one (141) items were translated identically. For the rest of the items, a mutual agreement was sought as to the most appropriate translation. The emerged Greek version was then given to two other bilingual scholars who translated the items back into English. To examine the content validity of the translated scale, all four individuals involved examined the original and the translated scales, checking whether each translated item and its corresponding original one was the same in meaning. After suggestions were considered by a panel of five stroke care experts, modifications to the questionnaire were made. The original NAQ was modified for use among stroke patients, and the wording was adjusted for a Greek context. A panel of experts, including two physiotherapists and two occupational therapists, assessed the relevance of the items to construct the questionnaire. After the expert panel meeting, modifications were made to the original NAQ, including the removal of items related to yard work, wheelchair transportation, retirement or nursing home considerations, and non-medical expenses deduction. The modified NAQ (mNAQ) used for data collection comprised 70 items in 7 domains (Mobility, Self-care, Home care, Communication, Rehabilitation/medical, Social/recreational, and Financial/government assistance) and was pre-tested and validated among ten patients with stroke (Supplementary Material S1). A pilot survey with 10 non-participants ensured content validity; no comprehension issues were reported, though fatigue arose from the 70-item questionnaire. The magnitude of each need is scored on a five-point scale from 1 = not a need to 5 indicating ‘[a] very large need’. The initial mNAQ scores ranged from 141 to 705, with higher scores indicating greater needs.

Furthermore, the National Institutes of Health Stroke Scale (NIHSS) and the Barthel Index (BI) were used. The NIHSS assesses post-stroke neurological condition via 11 factors [15]. The Barthel Index evaluates daily activity independence [16]. These measures collectively underpin the study’s robust methodology.

### 2.3. Data Analysis

AMOS (version 21.0) and SPSS (version 21.0) were used for statistical data processing, and more specifically: Descriptive analyses, Confirmatory Factor Analysis (CFA), Exploratory Factor Analysis (EFA), Cronbach’s alpha reliability analysis and Non-Parametric tests for independent samples.

## 3. Results

The study included 71 stroke patients with demographic characteristics detailed in Table 1. The data analysis revealed initial psychometric properties of the mNAQ were unsatisfactory. However, modifications led to a refined 31-item scale addressing communication, mobility, and social functioning. The validated Greek mNAQ effectively differentiated between acute and chronic stroke patients and those with minor and moderate strokes.

### 3.1. Sample’s Demographic Characteristics

Table 1 gives a full description of the sample’s demographic characteristics.

### 3.2. Scale Validation

In order to assess the psychometric properties of the mNAQ scale, first, item-to-total correlation value was estimated for each trait. Items with a corrected item-to-total correlation less than 0.30 should be discarded [17]. The results revealed that one item had less than the cut off criterion. Then, principal component analysis with oblimin rotation was performed. A three-factor pattern emerged after removing items that had high cross-loadings on multiple factors, indicating that they were not adequately measuring the intended construct. Removing such items enhanced the scale’s clarity and interpretability. Furthermore, multiple items in the original scale were highly correlated or measured similar aspects of the construct. Therefore, redundant items were eliminated to streamline the scale and reduce participant burden.

The EFA revealed that of the 31 remaining items, seventeen items exhibited significant loadings for the construct of “Mobility”, ten items for the construct of “Communication”, while four items showed significant loadings for the construct of “Social functioning”. These findings suggest that the resulting factorial patterns differ from the original mNAQ framework, though items removal enhanced the scale’s clarity and interpretability.

Another round of EFA was performed in the remaining 31 items and a clear three-factor structure was emerged. The new factor structure with the retain traits explained 81.78% of the total variance. All the items had strong loadings, and exceeded the 0.70 threshold [18]. The Bartlett’s test of sphericity (x^2^ = 4,648,334, df = 465) was significant (*p* < 0.001) and KMO measure was 0.731. Thus, the data satisfied the criteria for further analysis and indicated non-zero correlations [17]. The component correlation matrix ranged between 0.139 and 0.335 [18]. Table 2 shows the new purified scale with the three dimensions and thirty-one items.

### 3.3. Confirmatory Factor Analysis & Reliability Analysis

The validation of the scale involved confirmatory analysis using the generalized least square’s function, due to non-normality of the data distribution [19]. Two latent factors were assessed using various indicators and goodness-of-fit indices [20]. The overall model fit assessment indicated satisfactory fit indices (refer to Table 3). To evaluate reliability, both Cronbach’s alpha and composite reliability (CR) methods were employed. Cronbach’s alpha demonstrated adequate scores (0.984 and 0.822), and the composite reliability index ranged from 0.91 to 0.98, which were considered acceptable [21] (refer to Table 4). Subsequently, a validation process was conducted to assess the convergent and discriminant validity of the scale. Convergent validity was evaluated using two tests: (a) *t*-values, which ranged from 15.5 to 45.1 and met the appropriate threshold (≥±1.96) [22] and (b) Average Variance Extracted (AVE) for the latent variables, which exceeded the 0.50 cut-off criterion for each factor [23], with CR more than the acceptable level of 0.6 [24]. Discriminant validity was explored using the method proposed by Fornell and Lacker [21]; the square roots of AVE for all latent variables, were greater than the inter-correlations among the latent factors. Therefore, the validation of the scale was established.

### 3.4. Construct Validity and Nonparametric Tests

Afterwards, a second study was conducted to examine the concurrent and known-groups validity of the modified scale. To assess the concurrent validity of the mNAQ, we examined the relationship between the mNAQ scores and the BI scores. Spearman’s rho correlation was used, to determine if there is a significant association between the mNAQ scores and functional independence (BI). The negative correlation with BI scores (correlation coefficient = −0.500) indicates that higher rehabilitation needs are linked to lower functional independence. Then group comparisons were conducted to further investigate the known-groups validity of the scale. 

Τhe sample was divided into two groups based on the stroke phase of the participants (acute and chronic) and on the participants’ NIHSS scores (minor stroke and moderate stroke). Then, non-parametric tests were performed, to compare the mean mNAQ scores between these groups. A Mann-Whitney U test was used to find difference in rehabilitation needs between acute and chronic stroke patients and between patients who suffered a minor stroke and a moderate stroke. The level of significance was set at 0.05. Results of these analyses showed that: (1) only the distribution of needs in the “Mobility” factor differentiates across the two NIHSS groups significantly (sig. = 0.033) and (2) the distribution of needs in all the three factors of the scale, differentiate significantly across the categories of acute/subacute and chronic phase (communication needs: sig. = 0.029, mobility needs: sig. = 0.031, social functioning needs: sig. = 0.040).

## 4. Discussion

In this study, our primary aim was to validate the modified Needs Assessment Questionnaire (mNAQ) as a reliable and valid measure for assessing the needs of stroke patients within the Greek context. The exploratory factor analysis (EFA) was instrumental in revealing that the original mNAQ framework required refinement. Through a systematic process of eliminating items with low item-to-total correlations and cross-loadings, we successfully established a robust and interpretable three-factor structure: Mobility, Social functioning, and Communication. This refinement significantly improved the scale’s construct validity, ensuring that each item accurately measured its intended construct in a Greek patient setting. Moreover, the removal of redundant items streamlined the scale, enhancing its practicality and efficiency in clinical settings.

Our decision to eliminate certain items was motivated by the need to create a concise and practical questionnaire that could be easily administered to stroke patients and provide crucial pieces of information to healthcare professionals, as this was extensively discussed with the expert panel. The extensive length of the original scale posed a potential burden on patients, leading to response fatigue and reduced accuracy in their answers, as observed during our pilot study. By reducing the scale to 31 items, the adapted mNAQ became a more time-efficient and user-friendly tool, increasing its feasibility and acceptability for both patients and healthcare providers.

During the EFA process, items with low correlations and cross-loadings were identified and subsequently removed from the final version of the mNAQ. This rigorous approach ensured that the retained items had higher loadings on their respective factors, thereby enhancing the scale’s construct validity and accuracy in assessing specific rehabilitation needs. The confirmatory factor analysis (CFA) further corroborated the validity of the three-factor structure, while high internal consistency demonstrated by Cronbach’s alpha and composite reliability scores provided additional evidence of the reliability of the three constructs.

Our findings revealed significant differences in the needs reported by stroke patients based on the phase and severity of their stroke. Patients with moderate stroke were found to have more unmet mobility and motor control needs compared to those with minor strokes, while communication and social functioning needs were more pronounced among patients with moderate to severe strokes. These observations underscore the importance of tailoring interventions to address the specific needs of different patient groups, thereby optimizing rehabilitation outcomes.

Interestingly, acute-phase stroke patients perceived their needs in all three domains (mobility, communication, social functioning) as either partially met or non-existent. This suggests that acute-phase patients may have a different perspective on their rehabilitation needs, possibly influenced by their recent stroke event and initial stages of recovery. In contrast, chronic-phase stroke patients appeared to have developed a more comprehensive understanding of their rehabilitation needs over time.

Recognizing these differences is crucial for healthcare providers in prioritizing interventions that address individual patients’ challenges effectively. Targeted rehabilitation programs can lead to better functional outcomes for patients with moderate strokes, while addressing communication and social functioning needs can greatly benefit patients with more severe strokes [25].

In conclusion, the validated mNAQ, with its three-factor structure, serves as a valuable tool for assessing rehabilitation needs among stroke patients in the Greek context. Its psychometric properties enable targeted and personalized post-stroke care, accurately identifying and prioritizing the unique needs of stroke survivors.

## 5. Conclusions

The study successfully validated the mNAQ as a reliable and valid measure for assessing the rehabilitation needs of stroke patients in Greece. By refining the scale’s three-factor structure and demonstrating its psychometric properties, this study contributes to the optimization of post-stroke care delivery. The mNAQ is a valid and reliable measure that has the potential to improve post-stroke treatment and maximize rehabilitation results. The overall health and quality of life of stroke patients can be significantly improved by continuously refining and improving stroke care treatments based on the requirements that have been identified. Future studies can further enhance the delivery of stroke treatment, leading to better patient outcomes and overall stroke care, by examining the relationships between the stated needs and particular rehabilitation results.

## 6. Limitations and Future Research

However, certain restrictions must be recognized. The majority of the stroke patients in the sample were from a provincial area of Northern Greece, which may have limited the applicability of the results to a larger community of stroke victims. To improve external validity, future studies should strive to include a more representative and varied sample. Furthermore, additional cross-cultural validation could be required to determine the suitability of the measure in various cultural contexts. Future studies should examine the scale’s validity in various cultural contexts and look at how responsive it is to patients’ changing demands over time in order to improve stroke care and rehabilitation. The 31-item Greek modified requirements Assessment Questionnaire (mNAQ) has been successfully validated, highlighting its potential as a useful instrument for evaluating stroke patients’ requirements in the Greek setting. Through the distinction of three domains—communication, mobility, and social functioning demands—the mNAQ provides a more targeted comprehension of the particular needs of stroke patients. With the use of this information, healthcare professionals may more effectively customize interventions and support to meet the special requirements of stroke survivors.

## Figures and Tables

**Table 1:** Stroke patients’ sample demographic characteristics.

Sex	%	Phase of Stroke	%	Stroke Severity	%
Male	63.4	Acute/Subacute	79	Minor	78
Female	36.6	Chronic	21	Moderate	22
Age	%				
18–29	4.2				
30–39	7.0				
40–49	9.9				
50–59	18.3				
60–69	42.3				
70+	18.3				

**Table 2:** Factor Correlations for Exploratory Factor Analysis of mNAQ.

	Factor			Communalities
	Mobility	Social Functioning	Communication	
I need… 1. To be better able to move around in bed	0.812			0.713
2. To be better able to get in and out of bed or chairs	0.891			0.826
3. To be better able to get on and off of the toilet	0.857			0.824
4. To be better able to get in and out of a bath tub	0.891			0.901
5. To be better able to pick up things off the floor	0.828			0.793
6. To be better able to get in and out of a car	0.841			0.730
7. To be better able to use my wheelchair	0.928			0.862
8. To be better able to stand for long periods time	0.961			0.927
9. To learn how to get down and up from the floor	0.948			0.901
10. To be better able to walk in my home	0.867			0.758
11. To be better able to walk outdoors	0.910			0.828
12. To be better able to walk in crowded places	0.901			0.811
13. To be better able to dress my lower body (wear pants, shoes)	0.916			0.840
14. To improve my ability to dress my upper body (shirt)	0.879			0.775
15. Someone to help me to bathe or get dressed	0.913			0.842
16. Someone to make meals for me	0.877			0.777
17. Someone to help take care of my home	0.895			0.804
18. (More) contact with a pharmacist		0.832		0.794
19. (More) contact with a registered nurse		0.874		0.848
20. To be able to attend religious services or events		0.848		0.770
21. To be better able to participate in religious traditions in my home		0.868		0.852
22. To be better able to show affection			0.926	0.893
23. To be better able to be intimate with my spouse			0.930	0.904
24. To meet people and develop friendships			0.934	0.892
25. To better understand when people speak to me			0.946	0.906
26. To improve my ability to speak			0.820	0.763
27. To better use picture or writing to communicate			0.921	0.857
28. To be better able to make my needs known			0.836	0.860
29. To be better able to communicate in an emergency			0.951	0.908
30. To be better able to have a conversation with friends/family			0.858	0.900
31. To be better able to communicate for my banking and shopping			0.870	0.951
Eigenvalues	16.30	6.004	3.794	
% of variance	52.15	18.78	12.32	

Note: Extraction Method: Principal Component Analysis, Rotation Method: Oblimin with Kaiser Normalization.

**Table 3:** Model Fit Indices.

Model	Chi-Square	Df	*p*-Value	RMSEA	GFI	CFI	TLI	CMIN/df
Default model	640.8	431	0.0	0.1	0.4	0.3	0.2	1.5
Saturated model	0.0	0			1.0	1.0		
Independence model	746.7	465	0.0	0.1	0.3	0.0	0.0	1.6

**Table 4:** Confirmatory factor analysis of the scale, Mean scores & Standard Deviations.

Variables	Cronbach’s Alpha	CR	AVE	Mean Scores	S.D.
Mobility	0.984	0.984	0.749	2.0345	1.30571
Social Functioning	0.926	0.916	0.732	1.1127	0.55386
Communication	0.975	0.993	0.873	1.2831	0.84193

Note: CR = Composite Reliability; AVE = Average Variance Extracted, S.D. = Standard Deviations.

## Data Availability

The original contributions presented in the study are included in the article, further inquiries can be directed to the corresponding author.

## Supplementary Materials

The following supporting information can be downloaded at: https://www.mdpi.com/article/10.3390/healthcare12131274/s1, Supplementary S1: Modified Need Assessment Questionnaire.
