# Supplementary material for: Exploring Stroke Patients’ Needs: A Cultural Adaptation and Validation of the Modified Needs Assessment Questionnaire in a Greek Context
Source: Healthcare (Basel). 2024 Jun 26;12(13):1274. doi: 10.3390/healthcare12131274 (PMC11241691; doi:10.3390/healthcare12131274)
Supplement: Supplementary file 1 [file healthcare-12-01274-s001.zip › healthcare-3002627-supplementary.pdf]

healthcare

MDPI

Supplementary:

Modified Need Assessment Questionnaire

Instruction: With your help, we hope to better understand the needs of people who had a stroke episode. This study will help health professionals and communities more effectively meet the needs of people with stroke. We would appreciate your efforts to answer the following questions to the best of your ability.

For each statement please rate your current need by checking one of the five options to the right indicating the amount of need for each item e.g. "Not a need", to "A very large need".

1: Needs related to your ability to move from place to place

I need …	Not a need Or Need is already met	A small need	Moderate Need	A large need	A very large need	Not Applicable (N/A)	Check here if you currently perform this activity with assistance or equipment
1. To be better able to move around in bed							
2. To be better able to get in and out of bed or chairs							
3. To be better able to get on and off of the toilet							
4. To be better able to get in and out of a bath tub							
5. To be better able to pick up things off the floor							
6. To be better able to get in and out of a car							
7. To be better able to use my wheelchair							
8. To be better able to stand for long periods time							
9. To learn how to get down and up from the floor							
10. To be better able to walk in my home							
11. To be better able to walk outdoors							
12. To be better able to walk in crowded places							
13. To be better able to ascend and descend stairs							

2: Needs related to your ability to take care of yourself.

I need…	Not a need Or Need is already met	A small need	Moderate Need	A large need	A very large need	Not Applicable (N/A)	Check here if you currently perform this activity with assistance or equipment
1. To be better able to feed myself							
2. To be better able to chew and swallow food							
3. To control my saliva (drooling)							
4. To improve control of my bladder							
5. To improve control of my bowels							
6. To improve my ability to wash and bathe myself							
7. To be better able to dress my lower body (wear pants, shoes)							
8. To improve my ability to dress my upper body (shirt)							
9. To be able to drive							

3: Needs related to interacting and communicating with family, friends and others.

I need …	Not a need Or Need is already met	A small need	Moderate Need	A large need	A very large need	N/A	Check here if you currently perform this activity with assistance or equipment
1. To be better able to visit with family							
2. To be better able to show affection							
3. To be better able to be intimate with my spouse							
4. To meet people and develop friendships							
5. To better understand when people speak to me							
6. To improve my ability to speak							
7. To better use picture or writing to communicate							
8. To be better able to make my needs known							
9. To be better able to communicate in an emergency							
10. To be better able to have a conversation with friends/family							

11. To be better able to communicate for my banking and shopping.							

4: Needs related to services in my home or in the community

I need…	Not a need Or Need is already met	A small need	Moderate Need	A large need	A very large need	N/A	Check here if you currently perform this activity with assistance or equipment
1. Someone to help me to bathe or get dressed							
2. Someone to make meals for me							
3. Someone to help take care of my home							
4. More accessible public transport for people with disabilities (ie wheelchair accessible buses and cars)							
5. To have more control over decisions about my health							
6. More information on available community services							
7. To have more say regarding my care or community services I receive							
8. Help to decide whether I am still safe living at home alone							
9. Someone to help to take care of my family member							

5: Needs related to rehabilitation and medical services

I need …	Not a need Or Need is already met	A small need	Moderate Need	A large need	A very large need	N/A	Check here if you currently perform this activity with assistance or equipment
1. To see my doctor more frequently							
2. My doctor to make house calls							
3. To have (more) speech therapy regarding swallowing							
4. To have (more) speech therapy for communication							
5. To have (more) occupational therapy							
6. To have (more) physiotherapy							
7. (More) contact with a dietician/nutritionist							
8. (More) contact with a pharmacist							
9. (More) contact with a social worker							
10. (More) contact with a registered nurse							
11. Therapy that is closer to my own home							
12. Improved communication between my therapists and medical professionals							

6: Needs related to social and recreational activities

I need…	Not a need Or Need is already met	A small need	Moderate Need	A large need	A very large need	N/A	Check here if you currently perform this activity with assistance or equipment
1. To be able to read better							
2. To participate in sports activities better							
3. To be better able to do my hobbies							
4. To be able to attend religious services or events							
5. To be better able to participate in religious traditions in my home							
6. More convenient parking when I go out							
7. More convenient transportation to social activities							
8. More accessible transportation for long distance travel - eg. Special services for travelling by air, train							
9. To be able to get into buildings such as restaurants, theatres, arenas, religious buildings							
10. To have more convenient public washroom facilities							
11. (More) contact with a recreation therapist							

7: Needs related to financial assistance or government assistance

I need…	Not a need Or Need is already met	A small need	Moderate Need	A large need	A very large need	N/A	Check here if you currently perform this activity with assistance or equipment
1. Financial assistance to make renovations to make my home safer and more suitable for me							
2. Financial support to purchase equipment to help me take care of myself.							
3. Financial support to buy personal supplies like incontinence pads.							
4. Someone to help me access financial support I may be entitled to (eg. disability insurance)							

5.	Government recognition of my difficulties and needs	
